# Twin-twin transfusion syndrome - a University Hospital experience with intrauterine treatment

**DOI:** 10.1590/0100-6991e-20202671

**Published:** 2021-01-13

**Authors:** THAMYLE MODA DE SANTANA REZENDE, VIKTORIA WEIHERMANN, CAMILA GIRARDI FACHIN, RAFAEL FREDERICO BRUNS, ANDRÉ IVAN BRADLEY SANTOS DIAS

**Affiliations:** 1 - Federal University of Paraná, Department of Pediatric Surgery - Curitiba - PR - Brazil; 2 - Federal University of Paraná, Department of Gynecology and Obstetrics - Curitiba - PR - Brazil

**Keywords:** Fetofetal Transfusion, Laser Coagulation, Twins, Transfusão Feto-Fetal, Fetoscopia, Gravidez de Gêmeos

## Abstract

**Introduction:**

twin-to-twin transfusion syndrome (TTTS), defined by combination of polyhydramnios-oligohydramnios, is the most prevalent (5%-35%) of the abnormalities due to placental vascular anastomoses and the most lethal (80%-100% mortality) if untreated. Fetoscopic laser ablation of abnormal vasculature using the Solomon technique is the gold standard approach. It consists of interrupting the intertwin blood flow*.*

**Objectives:**

to present our initial experience at the Fetal Surgery Service of the Hospital de Clinicas of the Federal University of Parana (HC-UFPR) and to compare our results with those reported in the literature*.*

**Methods:**

we conducted a retrospective analysis of pregnancies who had undergone laser ablation, assessing data on Quintero’s staging, gestational age at diagnosis and at the time of the procedure, placental position, immediate post-procedure survival, and survival after the neonatal period. We then compared these data with the most recent data available in the literature.

**Results:**

we analyzed ten TTTS cases. The diagnosis was performed before the 26^th^ week of pregnancy (median 20.8 weeks) and treatment occurred in a median of 9.5 days later. The distribution by the Quintero’s staging was of three cases in stage II, five in stage III, and two in stage IV. In 50% of the gestations, at least one of the fetuses survived through the neonatal period.

**Conclusion:**

the treatment of TTTS in the HC-UFPR had a positive impact in the survival of the affected fetuses, although the results were worse than the ones reported in the literature, probably due to the delay in referencing the patients to our service, leading to a prolonged interval between diagnosis and treatment.

## RESUMO

**Introdução:**

a síndrome de transfusão feto-fetal (STFF), definida pela combinação polidrâmnio-oligohidrâmnio, é a mais prevalente (5 a 35%) das anormalidades associadas às anastomoses vasculares placentárias e tem a maior letalidade (80 a 100%) se não tratada. A ablação a laser destes vasos por via fetoscópica com a técnica de Solomon é o tratamento de escolha atual para a interrupção das anastomoses vasculares*.*

**Objetivo:**

apresentar a experiência inicial do Serviço de Cirurgia Fetal do Hospital de Clínicas da Universidade Federal do Paraná (HC-UFPR) e comparar nossos resultados com os da literatura*.*

**Métodos:**

foram revisados os prontuários de todas as pacientes submetidas ao procedimento de ablação a laser, sendo analisados os dados referentes ao estadiamento de Quintero, à idade gestacional ao diagnóstico e ao procedimento, à posição placentária, à sobrevida imediata pós-procedimento e sobrevida após o período neonatal. Os dados foram então comparados com os mais recentes disponíveis na literatura*.*

**Resultados:**

dez casos de STFF foram analisados. Todos diagnosticados antes da 26^a^ semana (mediana 20,79) e o intervalo diagnóstico-tratamento teve mediana de 9,5 dias. A distribuição pelo estadiamento de Quintero foi: três casos no estádio II, cinco casos no III e dois casos no IV. Em 50% das gestações pelo menos um dos fetos sobreviveu ao período neonatal*.*

**Conclusão:**

o tratamento da STFF no HC-UFPR impactou positivamente a sobrevida dos fetos acometidos. Entretanto, nossos resultados estão aquém dos reportados na literatura, possivelmente pelo demora no referenciamento das pacientes, com intervalo aumentado entre diagnóstico e tratamento.

## INTRODUCTION

Twin pregnancies often impose risks on mother and fetuses when compared with non-twin ones^1^. Monochorionic twin pregnancies are three to 10 times^2^ more susceptible to fetal morbidity and mortality^1^. One of the reasons for this is the almost always present vascular anastomoses^3^, which allow the shunting of blood between fetuses^4^ and can lead to the development of a group of pathophysiological conditions commonly referred to as “complicated monochorionic twins”^5^.

The forms of presentation of these conditions are: twin-twin transfusion syndrome (TTTS), whose incidence in monochorionic pregnancies varies between 5% and 35%; intrauterine growth restriction (IUGR), between 10% and 15%^5^; twin anemia-polycythemia sequence (TAPS), in 5%^6^; and twin reverse arterial perfusion sequence (TRAPS) - also known as an acardiac twin - in 1%^1^.

TTTS is defined ultrasonically by the combination of polyhydramnios in one amniotic sac and oligohydramnios in the other^2^, and is one of the most lethal perinatal complications, with mortality rates between 80% and 100% if left untreated^2^. The forms of treatment include amnioreduction, septostomy, selective feticide, and laser ablation of placental anastomoses via fetoscopy^2^. The latter is considered the current treatment of choice when TTTS occurs before 26 weeks of gestation^2^, as it displays the lowest neurological sequelae and the highest survival rates^7^ ^,^ ^8^. Treatment consists of preventing blood flow between the fetuses by coagulating the abnormal vessels that connect them.

Among the methods for laser coagulation, the Solomon selective technique has a higher survival rate for both fetuses and less persistence of uncoagulated vessels according to the literature^9^. Therefore, it was the technique chosen for implementation at the Hospital de Clinicas of UFPR (HC-UFPR), in 2016.

This work aimed to perform a retrospective analysis of patients diagnosed with TTTS who underwent laser ablation treatment at the Fetal Surgery Service of HC-UFPR, to compare different variables before and after intervention and fetuses’ survival with the results of the current literature.

## METHODS

This is a retrospective analysis study of patients diagnosed with TTTS treated by laser ablation of the placental vessels via festoscopy at the Fetal Surgery Service of the Hospital de Clinicas of the Federal University of Parana (HC-UFPR).

For the purposes of this study, we limited the series to patients treated by TTTS, with a confirmed ultrasonographic diagnosis at the HC-UFPR, and undergoing laser ablation via fetoscopy in this service. We excluded cases of selective intrauterine growth restriction and TRAPS, because although they are also related to the sharing of a placenta by the two fetuses and can be treated by laser ablation, they display different pathophysiology, prognosis, and complications. We excluded one of the TTTS cases from the analysis due to insufficient information in the medical records.

TTTS was classified ultrasonographically according to the Quintero’s staging, treatment being indicated for fetuses in stage II or higher and before the 26^th^ week of pregnancy, as recommended by the literature. Table 1 shows the Quintero’s staging.

**Table 1:** Quintero’s staging ^10^ .

Stage	Characteristics
I	Oligohydramnios in the donor sac and polyhydramnios in the recipient sac.
II	Absence of urine in the bladder of the donor fetus.
III	Abnormal blood flow on Doppler.
IV	Fetal hydrops.
V	Fetal death.

We used the Solomon technique for laser ablation of abnormal placental anastomoses via the fetoscopic route, given the greater evidence highlighting the superior effectiveness and less recurrence or inversion of transfusion between fetuses. This approach consists of the cauterization of visible anastomoses, followed by the interconnection of these sites by a laser cauterization line, from one end of the placenta to the other, demarcating the placental vascular equator^11^. The purpose of this last step is to completely separate the two parts of the placental chorionic surface^6^. This allows for a reduction in operative time and less damage to normal placental vessels, when compared with selective and non-selective methods used before the description of this technique^11^. For fetoscopic access, local anesthesia is performed over the entire thickness of the maternal abdominal wall, followed by a small 3 mm incision and ultrasound-guided insertion of the fetoscope in a placenta-free area^12^. Once the fetoscope is positioned, coagulation of vascular anastomoses occurs as described above^13^. The laser used in the service was the Deligth 1420 model (VYDENCE Medical^R^, Sao Carlos, SP, Brazil).

The variables analyzed in this study were gestational age at the time of diagnosis and treatment, Quintero’s staging and ultrasound estimation of fetal weights before and after intervention, survival of donor and recipient fetuses immediately after intervention, number of fetuses born alive, gestational age at birth, and survival in the neonatal period (28 days). The study was approved by the Ethics in Research Committee of the Hospital de Clinicas, Federal University of Parana, under number CAAE 65885917.2.0000.0096, report number 2.062.788, in May 2017.

For the statistical analysis, we calculated the medians, chosen as a parameter for grouping the data due to the small number of study subjects. We expressed mortality with the Kaplan-Meier curve. We used the PrismⓇ software v.7.0 (Graphpad, La Jolla, CA, United States). Lastly, we compared the results with those available in the literature.

## RESULTS

Fourteen patients were treated with laser ablation of the placental vessels via fetoscopy at the HC-UFPR Fetal Surgery Service between July 2016 and March 2018.

We excluded three of the cases because they had diagnoses other than TTTS, one of which was diagnosed only as selective IUGR, and the other two, as TRAPS. Of the 11 cases with confirmed TTTS, we excluded one from the analysis due to lack of data in the medical records.

Concerning the ten cases analyzed, the median gestational age at diagnosis was 20.8 weeks, the median gestational age at the time of treatment, 21.6 weeks, the interval between diagnosis and treatment varied between two and 15 days, with a median of 9.5 days (Table 2).

**Table 2:** Fetal ages at the times of diagnosis and procedure, in weeks; interval between diagnosis and procedure, in days.

	Fetal age at diagnosis (in weeks)	Fetal age at the time of the procedure (in weeks)	Interval between diagnosis and procedure (in days)
1	22.14	24.29	15
2	21.29	22.71	10
3	20.29	21.57	9
4	18.86	19.14	2
5	18.43	18.86	3
6	21.43	21.86	10
7	15.71	17.00	9
8	20.11	21.71	11
9	17.43	18.86	10
10	25.43	26.29	5
Median	20.79	21.64	9.5

**Figure 1:**
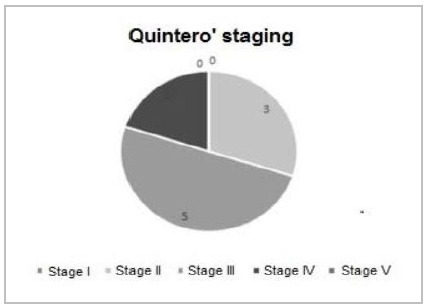
Quintero stage distribution.

The median weight of donor fetuses was 232g, while that of the recipient fetuses was 341g. Regarding the Quintero’s staging, we found stage I in no pregnancy, stage II in three, stage III in five, stage IV in two cases, and stage V also in none (Figure 1). The analysis of the placental position indicated three placentas in the anterior position, one in the posterior position, and the other six in lateral positions (Table 3).

**Table 3:** Data on fetuses at the time of diagnosis.

	Donor fetus weight (g)	Recipient fetus weight (g)	Placental position	Quintero’s Stage
1	548	778	Anterior	IV
2	428	548	Anterior	II
3	232	310	Posterior	III
4	161	206	Anterior	II
5	186	264	Lateral	III
6	290	465	Lateral	III
7	99	172	Lateral	III
8	465	ND	Lateral	II
9	225	372	Lateral	IV
10	ND	ND	Lateral	IV

Note: ND - no data available

**Figure 2:**
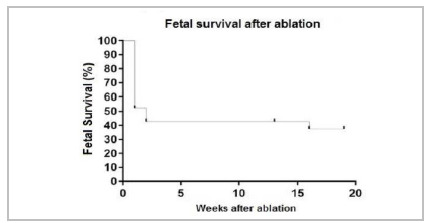
Kaplan-Meier curve of fetal survival after ablation.

Regarding fetal survival, in eight of the 10 pregnancies there were surviving fetuses in the immediate post-procedure period. In three of these, both fetuses survived, while in the other five, only one fetus survived, which results in 11 live fetuses out of 20 (55%) immediately after the procedure. Regarding survival until delivery, only one of the patients behaved differently, since both fetuses died two months after the procedure, resulting in a 45% survival rate (nine of the 20 fetuses were born alive). Finally, we observed survival after the neonatal period (28 days of life) in seven of the 20 fetuses (35%). In 50% of the cases (5/10) at least one of the fetuses survived after the neonatal period (Table 4 and Figure 2).

**Table 4:** Fetal survival immediately after the procedure, at birth, and after the neonatal period (28 days).

	Survival after the procedure	Survival during pregnancy	Survival after the neonatal period
	1	1	1
	1	1	1
	1	1	0
	2	0	0
	0	0	0
	0	0	0
	1	1	0
	1	1	1
	2	2	2
	2	2	2
Percent	55%	45%	35%

## DISCUSSION

 Anastomoses between placental vessels are present in 85% - 100% of all pregnancies of monochorionic twins, varying between arterio-arterial, arteriovenous, venous, and veno-arterial anastomoses. When anastomoses generate abnormal blood flow between fetuses, they can lead to diseases such as the twin-twin transfusion syndrome^9^.

Current scientific evidence points to laser ablation of placental vessels as the best method to reduce mortality by up to 80% and neurological sequelae in twins with TTTS^14^. The principle of this treatment is to interrupt vascular anastomoses to eliminate blood transfusion between the two fetuses^15^.

Previously used treatments, such as amnioreduction (repeated removal of amniotic fluid) and septostomy (perforation of the membrane between the twins) are less effective when compared with laser ablation via fetoscopy. Therefore, laser ablation is the method of choice for treating TTTS^7^ ^,^ ^8^. Supporting this statement, a systematic review^16^ published in 2005 gathered data from articles published between 1966 and 2004, indicating this technique as the best choice for reducing morbidity and mortality.

Laser ablation of placental vessels via festoscopy is a highly specialized procedure, performed in a few centers around the world^14^. The operation can be divided into two fundamental parts, the endoscopic identification of the placental vessels and the ablation of the anastomoses. These steps can be carried out based on selective or non-selective methods; the former identifies and coagulates only the placental vascular anastomoses, while the latter targets all vessels crossing the placental equator, regardless of whether they form anastomoses or not^17^. The Quintero’s technique fits into the selective method, and results in twice the survival of both twins (60%), versus only 30% of the non-selective, previously practiced methods^17^.

The more recently described Solomon technique, considered sequential (first with ablation of the vascular anastomoses and, finally, a cauterization line on the vascular equator), has a fetal survival rate between four and five times higher than that of Quintero^13^. This new technique is based on the fact that not all vessels can be seen by the endoscopic route; therefore, healthy areas between the vessels must also be coagulated in order to minimize the number of persisting anastomoses, which can be approximately 20% in the Quintero’s technique^17^.

Placental anastomoses can be superficial and bidirectional, such as arterio-arterial (AA) and venous-venous (VV), or deep and unidirectional, such as arteriovenous (AV) and veno-arterial (VA)^18^. The depth indicates whether the anastomosis - and not the entire vascular path - can be seen on the chorionic surface or occurs within the shared cotyledons, in the capillaries^19^. The direction of flow in the case of bidirectional vessels varies according to the pressure gradient between fetuses^18^.

This last characteristic allows AA shunts, with less resistance to flow, to compensate for the imbalance generated by arteriovenous anastomoses (AV) better than VAs, which display greater flow resistance. This leads to the belief that AA would have a protective effect on TTTS, while AV would be a necessary anatomical condition for the development of the syndrome^18^. AV is the most common type of anastomosis, identified in 75% of monochorionic placentas, whereas VV or AA are found in only 50%^4^.

The surgeon must be very well trained to identify these types of vessels during the procedure, in addition to having the appropriate equipment. For these reasons, this type of treatment is still quite restricted and rarely performed.

For all of the aforementioned reasons, fetoscopic laser ablation was the method chosen to be implemented in the Fetal Surgery Service of the Hospital de Clinicas of the Federal University of Parana in July 2016. Since the implantation until March 2018, 14 pregnancies had been treated that presented with abnormalities of vascular flow, eleven being TTTS. 

As noted in the results, the median fetal age at the time of treatment was 21.6 weeks. In one of the largest studies on the subject, carried out at the Fetal Medicine Center, in the United Kingdom, the median was 20.4 weeks^1^. This means that the treatment occurred late when compared to that reported by other authors^1^ ^,^ ^20^. This fact was probably due to the delay in referring the patient for the procedure. At HC-UFPR, the interval between diagnosis and the performance of the procedure had a median of nine days, while the literature recommends that this interval should be at most 48 hours, TTTS being a gestational emergency (personal communication by Dr D. Oepkes, in June 2016).

As for the Quintero’s staging, we observed three cases with stage II, five with stage III, and two with stage IV. Studies have shown that Quintero’s stage IV is related to lower survival^1^ ^,^ ^21^. In our series, of the two pregnancies classified as stage IV, three out of the four fetuses survived after the neonatal period. Thus, it is not possible to establish in our sample an association between the Quintero’ staging and fetal survival, probably due to our small number of cases.

Regarding fetuses survival, 55% survived the procedure, 45% were born alive, and 35% survived after the neonatal period, which demonstrates the impact of laser ablation via fetoscopy on the survival of these fetuses. According to the literature, survival would be zero to a maximum of 20% if left treated.

Another important data found in the literature is that the best results are obtained after about 61 procedures, or 3.4 years of experience, due to the learning curve^1^. Therefore, the greatest experience is closely related to the longest perinatal survival^1^. In this sense, it is worth mentioning that we report the initial experience of our service here. Yet, even in a short period, it is possible to notice a trend of improvement in the survival of the last treated cases in relation to the first, as shown in Table 4.

As for the limitations of this study, the main one is the small number of cases, which is partly due to the recent implantation of the technique in the service, as well as the small number of procedures performed due to financial and technical limitations. In addition, since this is a retrospective study, there is the inherent difficult in obtaining data from medical records.

Our analysis demonstrates that it is feasible to provide a highly complex treatment, such as fetoscopic ablation, for cases of TTTS in a public health service in a developing country, since the data showed an improvement in survival in relation to expectant management.

## CONCLUSIONS

The treatment of TTTS with laser ablation of placental vessels via fetoscopy at the HC-UFPR Fetal Surgery Service had a positive impact on the survival of the affected fetuses, with a better than expected result. Without treatment, this survival would be 0%-20%, and with a high incidence of sequelae.

However, these results of fetal survival in the service are still worse than those reported in the literature. One of the main factors responsible for this is probably the delay between diagnosis and treatment, which should be no longer than 48 hours, while at the HC-UFPR it was up to 15 days (median 9.5).
